# The impact of nationwide folic acid fortification on genetic variants associated with conotruncal heart defects

**DOI:** 10.21203/rs.3.rs-9984603/v1

**Published:** 2026-06-30

**Authors:** Sarah U. Morton, Enrique Mondragon-Estrada, Rui Qian, Omobola O. Oluwafemi, Kit Sing Au, Hope Northrup, Wendy K. Chung, A.J. Agopian, Tina O. Findley

**Affiliations:** Boston Children's Hospital; Boston Children's Hospital; Boston Children's Hospital; The University of Texas Health Science Center at Houston; The University of Texas Health Science Center at Houston; The University of Texas Health Science Center at Houston; Boston Children's Hospital; The University of Texas Health Science Center at Houston; The University of Texas Health Science Center at Houston

**Keywords:** congenital heart disease, conotruncal heart defect, folic acid fortification, folate

## Abstract

Folate deficiency is associated with an increased risk of conotruncal heart defects (CTHD), but interactions with genetic factors remain unclear. Our objective was to investigate genome-wide associations between genetic variants and birth before versus after universal folic acid fortification among children with CTHD and other heart defects. Genetic sequencing data were available through the Pediatric Cardiac Genomics Consortium. Sequencing data were aligned to the human reference genome (GRCh38/hg38) and jointly processed to ensure uniform variant detection and minimize batch effects. Analyses were restricted to individuals with European-inferred genetic ancestry. GWAS models were implemented to explore the association of common variants with fortification eras among all participants (n=1285), the subset of individuals with CTHD (n=534), and the remaining individuals with other heart defects (n=751). Functional enrichment was assessed using the Database for Annotation, Visualization and Integrated Discovery (DAVID). Among the full analytic group, eight loci had at least two nominally-enriched variants before compared to after fortification. Among the subset with CTHD, two variants located in *DHRS3* (rs7551703, OR 2.10, and rs6541043, OR 2.21) and four variants located in *PPARGC1β* were nominally enriched after fortification (OR 4.47–4.51). Enriched biological pathway terms were consistent with cardiac developmental processes. In summary, this study examined the association between folic acid fortification and genetic risk for CTHD and other heart defects and identified associations with fortification era that may suggest some shift in risk following fortification. Identified pathways suggest gene-nutrient interactions may modulate cardiac developmental pathways, underscoring the relationship between maternal folate status and cardiovascular development.

## Introduction

Congenital heart diseases are the leading cause of birth defect-associated morbidity and mortality, occurring in approximately one percent of live births (Zimmerman et al., 2020). Still, the etiology of most congenital heart diseases remains unclear. While a genetic condition is identified in approximately one-third of people with congenital heart diseases, most do not have a clear genetic association and are likely attributable to currently unknown complex etiologies (e.g., polygenetic risk, gene-environment interactions) (Hobbs et al., 2006, 2011; Mamasoula et al., 2013; Wang et al., 2013). Maternal dietary deficiencies during the early stages of pregnancy have been thoroughly investigated, including population studies and randomized control trials, demonstrating that periconceptional folic acid fortification is associated with decreased risk for the two most common birth defects, neural tube defects and conotruncal heart defects (CTHD) (Czeizel, 1993; Botto et al., 1996; Czeizel, Vereczkey and Szabó, 2015; Qu et al., 2020; Kolmaga et al., 2024). Based on these findings, the daily intake of prenatal vitamins containing folic acid is recommended for women of reproductive age, starting one to three months before conception. However, in many pregnancies, prenatal vitamin supplementation to prevent birth defects is often not started during this optimal window. Therefore, in 1998, the United States Food and Drug Administration mandated that grains and cereals be fortified with folic acid to prevent folate deficiency during the critical period of embryogenesis. While these measures significantly reduced the incidence of neural tube defects, a more modest impact occurred in congenital heart disease (Bedard et al., 2013; Czeizel et al., 2013), raising the likelihood of a more complex genetic-environmental relationship between folic acid and CTHD.

Folate plays a crucial role in one-carbon metabolism, nucleotide synthesis, and epigenetic regulation during embryonic development (Rosenquist, 2013). Its deficiency impairs the development of cardiac neural crest cells, resulting in structural defects of the cardiac outflow tract, valves, and septum, manifesting as CTHD (Tang et al., 2004; Taparia et al., 2007). However, genetic epidemiology studies investigating the relationship between variants in folate genes in CTHD have been inconclusive (Shaw et al., 2003; Goldmuntz et al., 2008; Zhu et al., 2012). A challenging aspect of these studies has been accounting for maternal folate deficiency and the relatively small study population. The fortification of grains and cereals with folic acid represents a population-level nutritional intervention that altered the prenatal environment, raising the possibility that it modified the penetrance or expressivity of some genetic risk variants. To our knowledge, no study has directly compared genetic variants among children with CTHD born before and after 1998 in North America, during which the blood levels of folate in the general population, including pregnant women, significantly increased due to folic acid fortification (Dietrich, Brown and Block, 2005).

Genome-wide association studies (GWAS) have identified multiple loci associated with congenital heart disease, yet many effect sizes are small and often inconsistent, with variability in the inclusion of subtypes contributing to study heterogeneity (Hu et al., 2013; Agopian et al., 2014, 2017; Mitchell et al., 2015; Hanchard et al., 2016; Lahm et al., 2021; Theis et al., 2023). Another explanation for this variability is unmodeled gene-environment interaction. Pre- and post-fortification birth cohorts present an opportunity to examine whether the distributions of genetic variation present among individuals with congenital heart disease differed across fortification eras. Our study objective was to address these previous limitations by utilizing a dataset of genomic sequencing data to explore the association of common genetic variants with pre- and post-fortification eras among children born with CTHD in the United States. The rationale for the study was to identify variants present in abundance before or after folic acid fortification, potentially representing risk alleles for CTHD that are sensitive to folate.

## Methods

### Study Subjects

Genomic sequencing data were accessed through the NCBI Database of Genotypes and Phenotypes (dbGaP) from the Pediatric Cardiac Genomics Consortium (PCGC) Study (phs001194.v2.p2) dataset. Details in the study design, patient population, and enrollment sites have been previously described (Pediatric Cardiac Genomics Consortium et al., 2013). Types of CTHD lesions in the case population were categorized based on expert opinion and the National Birth Defects Prevention Study (Botto et al., 2007). Our full analytic group included two subsets: individuals with CTHD and individuals with other congenital heart disease that did not have CTHD. CTHD was categorized using the PCGC-derived variable, which includes tetralogy of Fallot with or without pulmonary atresia, truncus arteriosus, D-transposition of the great arteries with or without ventricular septal defect, double outlet right ventricle, and interrupted aortic arch type B. Other cardiac defects (non-conotruncal) included septal defects (atrial septal defect and ventricular septal defect not classified as conotruncal), left-sided obstruction lesions, atrioventricular canal, total anomalous pulmonary veins, and single ventricle defects. Potential etiologic heterogeneity was suspected between these subsets, though both groups were recruited through the same multicenter study network, and individuals with other congenital heart diseases share many characteristics with individuals with CTHDs. Importantly, folic acid fortification has been shown to exert differential effects across congenital heart disease subtypes, with the strongest and most consistent associations observed for CTHD.

### Sequencing and Quality Control

All participants were sequenced through the PCGC using a centralized platform and harmonized quality control. Genome sequencing data were processed jointly to ensure uniform variant detection and minimize batch effects. Raw sequencing reads were aligned to the human reference genome (GRCh38/hg38). Joint variant calling was conducted across all samples, followed by joint genotyping. This approach allowed harmonized detection of single-nucleotide variants and small insertions/deletions (indels) across the combined cohort. Variants underwent standard quality control procedures, including variants that had genotype quality (GQ) > 20 and allele read depth (AD) > 10. Variants located within problematic genomic regions were removed using bedtools (Richter *et al*., 2020). Additional filters were applied to retain only bi-allelic SNPs with a minor allele frequency > 0.01, Hardy-Weinberg equilibrium p-value > 1×10^− 6^. To minimize population stratification and enhance genetic homogeneity, only individuals of predominantly European-inferred ancestry were included in the analysis to minimize the impact of ancestral differences between groups. Ancestry was inferred using EthSEQ (National Academies of Sciences, 2023). LD pruning was performed (50 kb window, step size = 5 variants, r^2^ > 0.2 threshold), and the pruned SNP set was used for principal component analysis.

### Analysis

The birth years of individuals in each cohort were used to categorize them into two time periods: before and after folic acid fortification, which was the primary exposure of interest. To account for a period of transition in the nationwide implementation of FDA policy, patients born in 1997 or earlier were grouped in the pre-fortification period, and patients born in 2000 or later were grouped in the post-fortification period. Patients with birth years of 1998 or 1999 were excluded as consumption of folic acid-fortified food during pregnancy was deemed uncertain. Thus, to investigate how folic acid fortification may have influenced allele frequencies in relation to CTHD, we first compared allele frequencies between all participants born before and after folic acid fortification among the full analytic group, including CTHD status as a covariate (GWAS Model 1). We then stratified this analysis by CTHD status, first restricting the analysis to individuals with CTHD (GWAS Model 2) and then restricting it to those with other congenital heart diseases (GWAS Model 3).

SNP-level genome-wide association analyses were performed using logistic-Firth hybrid regression under an additive genetic model with PLINK v2.0.0-a.6.22LM. Linkage disequilibrium score regression (LDSC) was used to estimate genomic inflation and confirm adequate control for population stratification, with LDSC intercepts guiding selection of the number of principal components to include. The top ten principal components were included as covariates in the GWAS model, as they provided optimal control for potential population stratification based on inspection of the LDSC intercepts. Suggestive statistical significance was defined with a threshold of p < 1 × 10^− 5^. All models were examined for variants in a predefined gene set, including *MTHFR, MTRR, MTR*, and related pathway genes (Lupo, Goldmuntz and Mitchell, 2010).

### Gene Set Analyses

To investigate biological pathways that may be involved in the etiology of CHDs, we conducted gene set enrichment analyses using the Database for Annotation, Visualization and Integrated Discovery (DAVID), version 6.8 (Huang, Sherman and Lempicki, 2009; Sherman et al., 2022). DAVID integrates multiple biological annotation databases, including Gene Ontology (GO), the Kyoto Encyclopedia of Genes and Genomes (KEGG), and Reactome, to evaluate whether predefined functional categories are overrepresented within a user-specified gene list. Enrichment analyses were conducted using a rank-based gene selection approach. For each of the three analytic models, genes were ranked according to their association Z-statistic, and the top 100 genes from each model were selected for downstream functional annotation. Using a fixed number of highly ranked genes ensured consistency across analyses while capturing genes showing the strongest evidence of association. Gene identifiers were uploaded to DAVID using Entrez Gene IDs, and enrichment analyses were performed using both the Functional Annotation Chart and Functional Annotation Clustering tools with default parameters (Huang, Sherman and Lempicki, 2009). Enrichment was evaluated across GO biological process, molecular function, and cellular component categories, as well as KEGG and Reactome pathway databases.

To minimize bias associated with gene set composition, enrichment analyses were conducted using a custom background gene list consisting of all genes included in the gene-level association analyses for each model. Statistical significance for enrichment was assessed using a modified Fisher’s exact test, and multiple comparisons were addressed using the Benjamini-Hochberg false discovery rate (FDR) adjustment implemented in DAVID (Huang, Sherman and Lempicki, 2009; Sherman et al., 2022). Annotation terms with FDR-adjusted p-values < 0.05 were considered statistically significant. Enrichment scores for annotation clusters were calculated as the −log10 of the geometric mean of the p-values for the terms within each cluster (Huang, Sherman and Lempicki, 2009; Sherman et al., 2022). Enrichment results were prioritized using nominal p-values < 0.05 together with fold enrichment (FE) ≥ 2, consistent with previously described exploratory enrichment strategies (Huang, Sherman and Lempicki, 2009; Raggi et al., 2020; Li et al., 2021). Functional annotation clusters with enrichment scores greater than 1.3 (approximately corresponding to a mean p-value < 0.05) were considered to represent meaningful enrichment signals (Huang, Sherman and Lempicki, 2009; Hazelett et al., 2014). For visualization, enriched annotation terms meeting the criteria of nominal p < 0.05 and FE ≥ 2 were manually reviewed to remove redundant entries. Terms representing overlapping biological concepts, including parent-child GO relationships or categories supported by highly overlapping gene sets, were consolidated to improve clarity. Enrichment results were summarized using dot plots, with annotation terms shown on the y-axis, −log10 transformed nominal p-values on the x-axis, and dot size reflecting the number of genes contributing to each enriched term.

## Results

**Figure 1:**
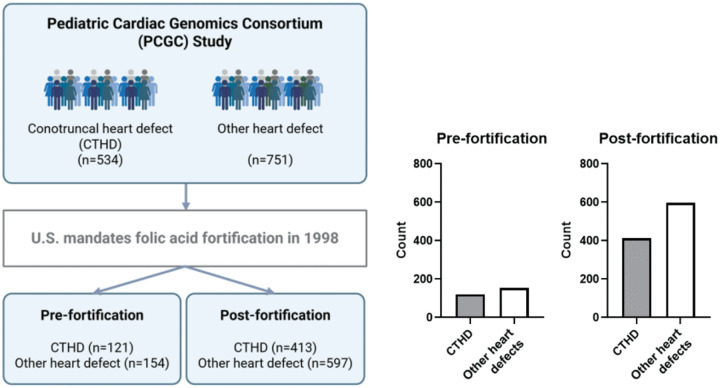
Consort diagram of study participants and genotype data available separated by folate era by European-inferred ancestry.

Across the entire PCGC cohort, tetralogy of Fallot was the most common subtype of CTHD (n = 1,708; 54.7%), followed by D-transposition of the great arteries (n = 845; 27.1%), double outlet right ventricle (n = 244; 7.8%), combined double outlet right ventricle with D-transposition of the great arteries (n = 70; 2.2%), truncus arteriosus (n = 203; 6.5%), and interrupted aortic arch, type B (n = 51; 1.6%). Following quality control measures, genotype data were available for 778 individuals with CTHD and 1,111 participants with other congenital heart diseases. After restricting to participants of European-inferred ancestry, 534 participants with CTHD (pre-fortification, n = 121; post-fortification, n = 413) and 751 participants with other heart defects remained (pre-fortification, n = 154; post-fortification, n = 597) (Fig. 1).

**Figure 2:**
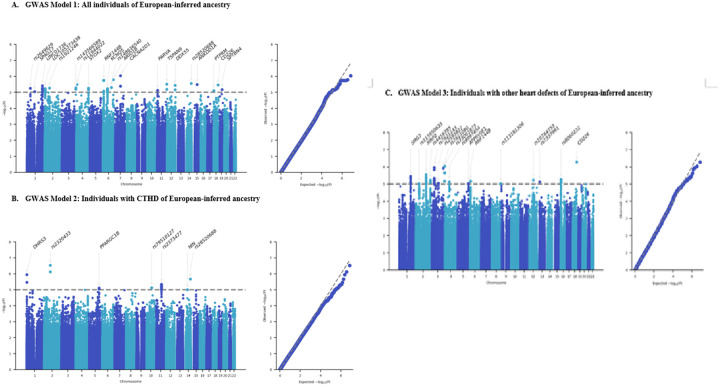
Manhattan plots showing genome-wide association results for children of European-inferred ancestry born before vs. after folic acid fortification and their respective quantile-quantile (QQ) plot and genomic control inflation factor (λ_GC_). A) GWAS Model 1 includes all individuals, including CTHD status as a covariate. B.) GWAS Model 2 includes individuals with CTHD. C.) GWAS Model 3 includes individuals with other heart defects. The horizontal line denotes the threshold for suggestive significance at p = 1 × 10^−5^. Variants above this line show nominal association but do not meet the conventional genome-wide significance threshold.

The summary results from the three GWAS models are shown as Manhattan plots, along with the GWAS quantile-quantile plot, in Fig. 2A–C. Overall, genomic inflation was minimal with the first 10 principal components included as covariates (λGC = 1.009), and LDSC indicated inflation was attributable to polygenicity rather than confounding (intercept = 1.006). Comparable genomic control was observed across all models. Among all study participants (Model 1), 94 variants were identified as nominally enriched (p < 10^− 5^) when comparing all participants born before and after folic acid fortification (Supplementary Table 1). Most variants identified in Model 1 were enriched prior to folic acid fortification (OR < 1), suggesting a shift whereby these genetic variants were present among individuals with congenital heart disease less frequently following fortification implementation. Nominally significant variants in Model 1 included multiple variants near *LINC01736* (rs67537171, p = 6.47 × 10^− 6^, OR 0.49). In Model 2 (individuals with CTHD), 26 variants were identified in individuals born before and after folic acid fortification. Variants located in *DHRS3* (rs7551703 and rs6541043) were nominally enriched among post-fortification births (p = 3.43 × 10^− 6^ and 1.13 × 10^− 6^, respectively), suggesting at least two-fold higher odds of the allele being present after fortification (OR 2.10 and 2.21, respectively). Similarly, variants localized to *MIR378A*, an intronic miRNA within the *PPARGC1β* locus (rs251462, rs32589, rs32585, rs32584) were associated with at least a four-fold increase in the odds of occurrence in the post-fortification group (p-values 7.75–8.84 × 10^− 06^, OR 4.47–4.51). In Model 3 (individuals with other heart defects), 59 variants were identified as nominally enriched in individuals born before or after folic acid fortification. Among these, eight variants within *MIR548A1HG* and one variant within *NK1–1* showed nominal associations in Models 1 and 3, and all were nominally enriched among pre-fortification births. The overlap across models suggests that these variants are not restricted to CTHD but may reflect genetic susceptibility shared across broader cardiac phenotypes or driven by non-conotruncal heart defects. Of note, no variants classically associated with folate transport or one-carbon metabolism were detected in any of the models.

**Figure 3:**
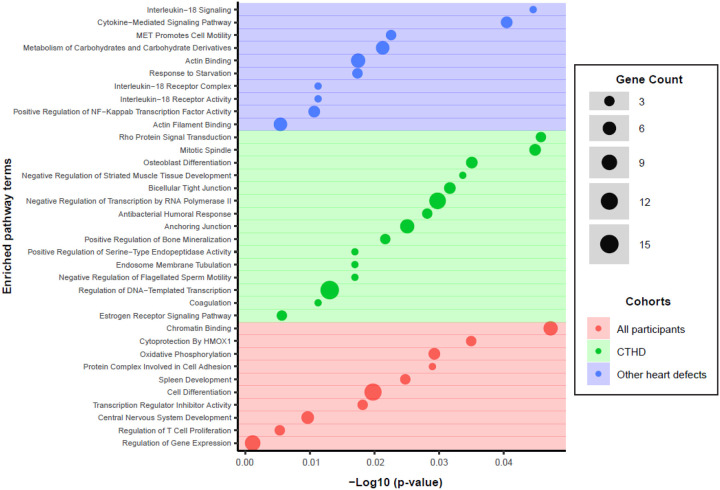
DAVID functional enrichment dot plot across the same cohorts used in GSEA models. Each cohort show distinct enriched terms. Dot size reflect the number of genes in the term. Enrichment scores for annotation clusters were calculated as the −log10 of the geometric mean of the nominal p-values.

Functional enrichment was assessed with DAVID across the same three analytic groups and results are summarized by dot plots in Fig. 3. Several biological themes reached nominal significance (unadjusted p < 0.05) for each group. Each group exhibited a distinct set of enriched terms (Fig. 3), further suggesting that phenotype-specific shifts in cardiogenetic pathways may have occurred following fortification. Top enriched biological pathway terms in the individuals with CTHD (Model 2) included Rho protein signaling, cytoskeletal dynamics, cell-cell junctions, transcriptional regulation, and intracellular trafficking. These pathway terms are consistent with core developmental processes relevant to heart formation. In individuals with other heart defects (Model 3), top enriched pathway terms included chromatin binding, regulation of gene expression, and oxidative phosphorylation, relevant for broader regulatory and metabolic processes. However, given that p-values were not corrected for multiple testing, these results should be interpreted as hypothesis-generating rather than confirmatory. A list of all enriched pathway terms and p-values for each group are provided in Supplemental Table 2.

## Discussion

Our genome-wide association analysis identified loci in dehydrogenase/reductase 3 (*DHRS3*) and peroxisome proliferator-activated receptor gamma coactivator 1-beta (*PPARGC1β*) among individuals with CTHD that showed differential association with the period of implementation of folic acid fortification in the United States. Among the full analytic group (Model 1) and among individuals with other congenital heart diseases (Model 3), a greater number of variants were associated with pre-fortification births compared to among those with CTHD (Model 2), suggesting that observed differences were not CTHD-specific or even lesion-specific.

The broader distribution of nominally significant variants among all participants (Model 1) and individuals with other congenital heart diseases (Model 3) may reflect population-wide genomic responses to environment and nutritional shifts following folic acid fortification. Folic acid fortification potentially altered one-carbon metabolism across the entire population during the post-fortification era, influencing methylation and possibly unmasking subtle allele frequency differences. Such widespread changes could generate multiple modest association signals when all participants are analysed together, consistent with a global environmental exposure effect. Interestingly, most of the variants identified in all participants and individuals with other congenital heart diseases analyses were more frequent in pre-fortification births and may have become rarer after fortification. This directionality suggests population-level response to increased folic acid exposure. This framework underscores the potential impact of folic acid fortification on population phenotype shift.

For instance, the nominal association of *MIR548A1HG* and *NKX1–1* across both Model 1 and 3 indicates that these loci are unlikely to be specific to CTHD and instead represent shared genetic risk factors for broader cardiac phenotypes. Although *NKX1–1* has not been established as a canonical gene in cardiac development, its identification in our pre-fortification GWAS models is biologically intriguing. *NKX1–1* is an NK-family homeobox transcription factor, a class of genes involved in developmental regulation (Simon and Lufkin, 2003). Prior cell-free fetal transcriptomic studies have reported its increased expression in the amniotic fluid from fetuses with trisomy 18 and trisomy 21 (Koide et al., 2011), genetic aneuploidies characterized by congenital heart defects. Together, these observations suggest that *NKX1–1* may represent a folate-sensitive regulatory locus but should be interpreted as hypothesis-generating.

In contrast, the more limited number of associated variants among affected individuals suggests that CTHD risk is shaped by a narrower set of loci that interact directly with nutrient-sensitive developmental pathways, although the reason that fewer variants reached nominal significance within participants with CTHD (Model 2), may also relate more to the CTHD group being smaller and therefore having more limited power. Regardless, the CTHD analysis indicated that carriers of *DHRS3* and *PPARGC1β* alleles were more likely to have been born after folate fortification, and these genes share biological pathways essential for cardiac morphogenesis and metabolic adaptation, which points toward biologically plausible mechanisms. These findings may support a model in which fortification broadly modified the genomic landscape of folate-responsive loci, while persistent associations in *DHRS3* and *PPARGC1β* represent targeted, biologically focused mechanisms. *DHRS3* is an evolutionarily conserved enzyme that plays a critical regulatory role in embryonic retinoid homeostasis, catalyzing the reduction of all-trans-retinaldehyde to all-trans-retinol, thereby maintaining appropriate intracellular retinoic acid levels during development (Belyaeva et al., 2015). Excessive and deficient amounts of retinoic acid, the active metabolite of vitamin A, and retinoid therapies are well-known teratogens associated with increased risk of spontaneous abortions and birth defects, including congenital heart disease (Enneth et al., 1995). Retinoic acid induces birth defects in animals, namely myelomeningocele, anencephaly, and CTHD (Cohlan, 1954; Yasui et al., 1995; Danzer et al., 2005; Cipollone et al., 2006). In the genetic model, ablation of the orthologous gene in multiple species increases retinoic acid levels, with *Dhrs3*^*−/−*^ embryos presenting with defects in cardiac outflow tract formation and atrial and ventricular septation, along with defects in skeletal, and craniofacial development (Danzer et al., 2005; Billings et al., 2013). In humans, inherited variants in *DHRS3* have been reported in families presenting with phenotypic features of craniosynostosis, facial dysmorphism, congenital heart disease (observed in 4 out of 5 affected individuals), and scoliosis (Hashimoto et al., 2025). Importantly, maternal folate deficiency is independently linked to an increased risk of neural tube defects and congenital heart disease (Czeizel, 1993; Czeizel et al., 2013) and experimental models demonstrate that folic acid supplementation can rescue neural tube defects, cleft palate, and congenital heart disease induced by retinoic acid or its antagonist (Reynolds, Schaalje and Seegmiller, 2003; Cipollone et al., 2009; Wu et al., 2020). These studies have been instrumental in establishing the role of prenatal vitamin supplementation in birth defect prevention.

PPARGC1β, also known as PGC1β, belongs to a family of transcriptional coactivators that regulate cardiac development and mitochondrial gene expression (Rowe, Jiang and Arany, 2010; Murphy et al., 2021), particularly in models of heart failure (Sihag et al., 2009). While PGC1α has been studied more extensively, other PPARs, including PGC1β, have been shown to share overlapping functions in metabolic regulation and mitochondrial biogenesis. The role of PGC1β is best established during the postnatal period when it activates the metabolic and functional maturation of the heart. In loss-of-function studies, PGC1αβ1^−/−^ mice display structurally normal but smaller hearts and exhibit conduction abnormalities such as bradycardia and heart block (Lai et al., 2008). PGC1β appears to be especially important during late fetal and perinatal cardiac development and likely interacts minimally with maternal folate status during early embryogenesis. However, the observed association could reflect a survival bias introduced by temporal changes in clinical outcomes. Over the study period, advances in pediatric intensive care and pediatric cardiac surgery have markedly improved survival among children with CTHD (Oster et al., 2013). It is therefore plausible that genetic variants predisposing to heart failure, such as those in *PGC1β*, may have become more prevalent in surviving cohorts, effectively altering the population structure of children with CTHD in the post-fortification era.

Strengths of our association study on the impact of folate supplementation on CTHD include the integration of the United States folic acid fortification policy into the study design. We accounted for the phased implementation and adoption of fortification during pregnancy by incorporating a two-year washout period, thereby improving classification of the two exposure groups. Additionally, we utilized existing high-quality genomic data from a well-phenotyped cohort available through the PCGC, enabling analysis of a relatively large population with CTHD to maximize statistical power. A key design consideration was the use of participants with other congenital heart diseases from the same multicenter study network as the reference group, rather than unaffected controls. This approach was intended to minimize bias related to healthcare access and ascertainment. Finally, rigorous QC measures, including restriction to individuals of European-inferred ancestry, minimized population stratification and reduced confounding due to genetic heterogeneity.

Limitations of our study include potential phenotypic heterogeneity among all of our analytic groups, as specific lesions may differ in their underlying genetic or environmental etiologies, which is a common limitation in GWAS of congenital heart disease. In this exploratory study, we also reported nominal associations that need confirmation in larger independent cohorts as they become available. The use of a less stringent significance threshold increases the possibility of false-positive associations, and thus, the results should be interpreted cautiously and validated in independent cohorts. The absence of detailed, individual-level data on periconceptional maternal diet and supplement use prevented assessment of individual-level folate exposure, and therefore, the effects of fortification may vary according to the baseline nutritional status of the individual. In fact, observed differences between pre- and post-fortification cohorts may have partially reflect unrelated secular trends over time, such as improved prenatal detection and survival of complex lesions or population changes in maternal characteristics (e.g., obesity) and behaviors (e.g., smoking) over time rather than directly relating to fortification policy. Although replication in independent populations is warranted, the PCGC remains one of the largest of United States congenital heart disease cohorts with genomic data. Additionally, restricting analyses to individuals of European-inferred ancestry reduces confounding from population stratification but limits the generalizability of findings to other ancestral groups. Our study also focused on the offspring genetic effect rather than the maternal genetic effect, and future work is needed to better understand potential changes in associated maternal variants following fortification. Despite these limitations, the study offers valuable insights into potential shifts in pathways and genetic factors among individuals with CTHD and other congenital heart disease following the era of fortification.

## Conclusion

In summary, this study integrates genomic and temporal data to provide insight into how folic acid fortification policy may have influenced genetic risk for CTHD. Our study captures this historical policy milestone as a natural experiment to examine folate-genotype interactions. Importantly, these findings extend beyond a historical policy context as mandatory folic acid fortification remains absent in many countries, meaning the gene-nutrient interactions identified here may reflect nutritional conditions still experienced by the global majority. By leveraging a large, well-phenotyped cohort, we identified potential gene-nutrient interactions that may modulate cardiac developmental pathways. These findings highlight the complex interplay between maternal folate status and embryonic retinoid levels in shaping cardiovascular development. Continued investigation into nutrient-gene interactions may inform targeted prevention strategies and refine our understanding of how population-level nutritional policies can reshape the genetic architecture of disease susceptibility.

## Supplementary Material

Supplementary Files

This is a list of supplementary files associated with this preprint. Click to download.

- floatimage1.png
- SupplementalTables.docx

## Data Availability

The datasets generated during and/or analysed during the current study are available in the Genotypes and Phenotypes (dbGaP) repository, https://dbgap.ncbi.nlm.nih.gov/beta/study/phs000571.v7.p3/#study.
